# Computer-Aided Drug Design of Novel Derivatives of 2-Amino-7,9-dihydro-8H-purin-8-one as Potent Pan-Janus JAK3 Inhibitors

**DOI:** 10.3390/molecules28155914

**Published:** 2023-08-06

**Authors:** Abdelmoujoud Faris, Ibrahim M. Ibrahim, Omkulthom Al kamaly, Asmaa Saleh, Menana Elhallaoui

**Affiliations:** 1 LIMAS, Department of Chemical Sciences, Faculty of Sciences Dhar El Mahraz, Sidi Mohamed Ben Abdellah University, Fez 30000, Morocco; melhallaoui@yahoo.fr; 2 Biophysics Department, Faculty of Science, Cairo University, Cairo 12613, Egypt; ibrahim_mohamed@cu.edu.eg; 3 Department of Pharmaceutical Sciences, College of Pharmacy, Princess Nourah Bint Abdulrahman University, P.O. Box 84428, Riyadh 11671, Saudi Arabia; omalkmali@pnu.edu.sa (O.A.k.); asali@pnu.edu.sa (A.S.)

**Keywords:** MM/GBSA, 3D-QSAR, drug discovery, JAK3, computational modeling, cancer, rheumatoid arthritis

## Abstract

Rheumatoid arthritis (RA) remains one of the most prevalent autoimmune diseases worldwide. Janus kinase 3 (JAK3) is an essential enzyme for treating autoimmune diseases, including RA. Molecular modeling techniques play a crucial role in the search for new drugs by reducing time delays. In this study, the 3D-QSAR approach is employed to predict new JAK3 inhibitors. Two robust models, both field-based with R^2^ = 0.93, R = 0.96, and Q^2^ = 87, and atom-based with R^2^ = 0.94, R = 0.97, and Q^2^ = 86, yielded good results by identifying groups that may readily direct their interaction. A reliable pharmacophore model, DHRRR1, was provided in this work to enable the clear characterization of chemical features, leading to the design of 13 inhibitors with their pIC_50_ values. The DHRRR1 model yielded a validation result with a ROC value of 0.87. Five promising inhibitors were selected for further study based on an ADMET analysis of their pharmacokinetic properties and covalent docking (CovDock). Compared to the FDA-approved drug tofacitinib, the pharmaceutical features, binding affinity and stability of the inhibitors were analyzed through CovDock, 300 ns molecular dynamics simulations, free energy binding calculations and ADMET predictions. The results show that the inhibitors have strong binding affinity, stability and favorable pharmaceutical properties. The newly predicted molecules, as JAK3 inhibitors for the treatment of RA, are promising candidates for use as drugs.

## 1. Introduction

The Janus family of kinases (JAKs) and STAT transcription factors mediate signaling pathways that are important in cellular activities, such as growth, differentiation and homeostasis. The abnormal activation of this signaling cascade has been linked to the pathophysiology of autoimmune disorders, such as rheumatoid arthritis and cancer [1,2].

JAK3 is a component of JAKs involved in the signaling of interleukin-2 (IL-2) and other cytokines that control immune responses. It is noteworthy that mutations in JAK3’s Cys_909_ residue may result in the constitutive activation of the kinase, which can contribute to the development of autoimmune disorders [3,4]. JAK3 inhibition is a viable therapeutic method for the treatment of RA and other autoimmune illnesses. JAK3 inhibitors have been shown in preclinical and clinical research to be effective in lowering RA symptoms and slowing disease progression. Furthermore, because JAK3 is involved in cell proliferation and death, it has the potential to be used in cancer therapy. JAK3 inhibitors, in particular, have been demonstrated to decrease the production of pro-inflammatory cytokines, such as IL-2, IL-6 and IL-17, all of which contribute to the pathophysiology of rheumatoid arthritis. Furthermore, these inhibitors can limit the synthesis of growth factors and cytokines, which are necessary for cancer cell proliferation and survival, thereby reducing cancer development [1,5,6,7,8,9,10].

**Figure 1:**
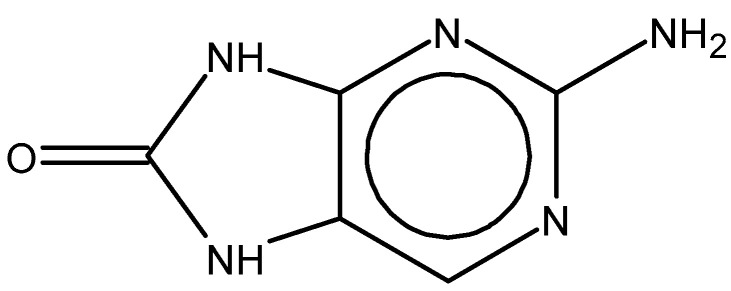
2-Amino-7,9-dihydro-8H-purin-8-one.

Importantly, the discovery of the crystalline structure of JAK3 (*ID: 4Z16*) has shown the relevance and critical function of the Cys_909_ residue. This particular residue is found in the protein’s active region and is required for catalytic activity. Cys_909_ mutations or modifications have been shown in studies to have a significant influence on JAK3 function [11,12,13]. Certain JAK3 inhibitors create covalent connections with the Cys_909_ residue, increasing their affinity and selectivity for this enzyme. This important interaction between Cys_909_ and JAK3 inhibitors can be used to develop more selective and powerful medicines for RA therapy [14]. Furthermore, understanding the placement and significance of Cys_909_ in the structure of JAK3 allows for a better understanding of the underlying molecular processes involved in this protein’s inhibition. This information can be used to drive the rational design of new inhibitory compounds that selectively target this active area, enhancing the effectiveness and selectivity of medications in development [15]. 2-amino-7,9-dihydro-8H-purin-8-one is a compound that has demonstrated its efficacy in the treatment of several diseases (Figure 1). In particular, it has been identified as a potential inhibitor of Janus kinases (JAKs) and as an anticancer agent [16,17,18].

**Figure 2:**
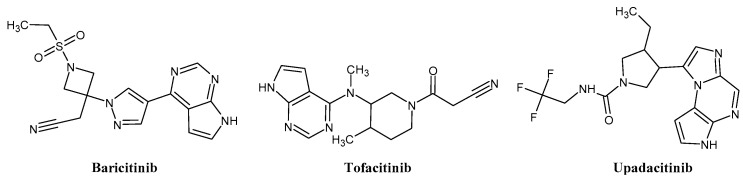
Two-dimensional representations of baricitinib, tofacitinib, and upadacitinib.

The FDA has approved the pharmaceuticals baricitinib, tofacitinib (Figure 2) and upadacitinib as members of the Janus kinase (JAK) inhibitor family of therapies [19,20,21]. These drugs target specific types of enzymes, known as kinases, that play a critical role in the immune system’s response to inflammation. Tofacitinib primarily inhibits JAK3 and to a lesser extent JAK1 and JAK2. By blocking JAK3, tofacitinib reduces inflammation and helps to slow down the progression of the disease. In contrast, Baricitinib primarily inhibits JAK1 and JAK2, which are involved in signaling pathways that regulate immune responses and inflammatory processes. Upadacitinib selectively targets JAK1, which is involved in regulating the differentiation and function of several immune cells. Tofacitinib was chosen for this study because it specifically targets JAK3. Tofacitinib works very well to treat rheumatoid arthritis by blocking JAK3 [22], a vital regulator of immune cell differentiation and proliferation. Tofacitinib has been linked to several severe adverse effects in addition to its efficacy. New inhibitors with novel designs are being created as a result, with the potential to provide improved effectiveness and fewer adverse effects.

The process of finding new drugs has become more and more dependent on computer-aided drug discovery (CADD). By swiftly finding prospective medication candidates from vast libraries of chemical compounds, it enables the process to be accelerated. By locating the most promising compounds more quickly and precisely than conventional approaches, CADD also aids in lowering costs and hazards related to clinical trials. Scientists may logically and effectively design and optimize medications using CADD, which may result in the development of novel treatments for diseases that were once incurable [23,24]. In this study, we employ a quantitative three-dimensional quantitative structure–activity relationship (3D-QSAR) and pharmacophore-based approach to identify key features crucial for the selective inhibition of JAK3 of novel derivatives of 2-amino-7,9-dihydro-8H-purin-8-one [25]. Additionally, absorption, distribution, metabolism, excretion and toxicity (ADMET) studies were conducted to predict the pharmacokinetic and pharmaceutical properties of candidate ligands, encompassing solubility, permeability and metabolic stability, which are pivotal for drug efficacy and pharmacokinetics [26,27]. Furthermore, we investigate covalent interactions between JAK3 and ligands through Cys_909_, recognizing its vital role in affinity and binding during cancer and RA cell recognition [28,29,30]. To identify potentially potent molecules for selective JAK3 inhibition, molecular dynamics (MD) simulations and molecular mechanics-generalized Born surface area (MM/GBSA) calculations were performed [31]. Notably, MD simulations were conducted over a 300 ns to assess the stability of ligand–JAK3 complexes.

## 2. Results and Discussion

### 2.1. Three-Dimensional-QSAR Models

#### 2.1.1. Analysis Statistics (Field-Based and Atom-Based)

**Figure 3:**
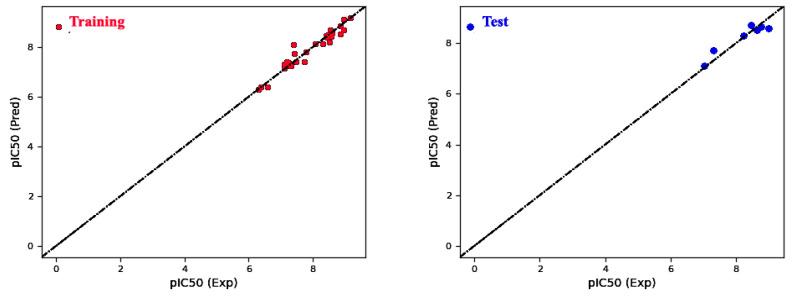
Graphical comparison of predicted versus actual pIC_50_ values for field-based models in the training and test sets.

The selection of Model 4 (Table 1) for the field-based approach is justified based on several factors. Firstly, Model 4 exhibited the highest coefficient of determination (R^2^) among all the models and demonstrated a high $R_{CV}^{2}$, indicating strong predictive capacity and low variance. Furthermore, Model 4 displayed a lower root-mean-square error (RMSE) compared to the other models (Figure 3), signifying improved accuracy in predictions. Upon examining the contributing factors within Model 4, it becomes evident that the Gaussian steric term contributes the most to the predicted biological activity, followed by the Gaussian hydrophobic term and the Gaussian H-bond acceptor (Table 2).

The model’s ability to predict the activity of new compounds was evaluated using Q^2^, in addition to the criteria R^2^ and $R_{CV}^{2}$, and was a key factor in the final selection process (Figure 2). Furthermore, to ensure precise and dependable predictions, the quality of data and other factors, such as model robustness, simplicity and interpretability, must also be taken into consideration. In the case of our model, both field-based (with Q^2^ of 0.87) and atom-based (with Q^2^ of 0.86) methods were evaluated, and both demonstrated good predictive power.

These findings highlight the critical importance of steric, hydrophobic and hydrogen bonding interactions in governing the biological activity of the investigated molecules. In summary, Model 4 was chosen for the field-based method due to its exceptional predictive performance, primarily attributed to the significant roles played by steric, hydrophobic and hydrogen bonding terms in forecasting biological activity. Similarly, Model 4 (Table 3) was selected for the atom-based method for various reasons. 

**Figure 4:**
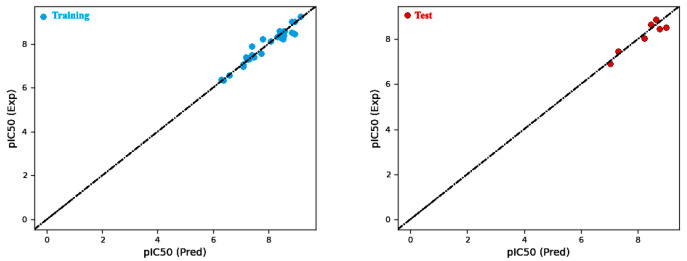
Comparison of predicted versus actual pIC_50_ values for atom-based models in the training and test sets.

Firstly, Model 4 demonstrated the highest coefficient of determination (R^2^) among all the models and exhibited a high R^2^_CV_, indicating robust predictive capability and low variance. Additionally, Model 4 yield a lower RMSE compared to the other models (Figure 4), thereby indicating an enhanced prediction accuracy. Upon the analysis of the factors within Model 4, it is observed that the hydrophobic/nonpolar term contributes the most to the predicted biological activity, followed by the H-bond donor and electron-withdrawing terms (Table 3 and Table 4). These findings emphasize the vital role of hydrophobic and hydrogen-bonding interactions in determining the biological activity of the studied molecules. In summary, Model 4 was chosen for the atom-based method due to its superior predictive performance, primarily driven by the substantial influence of hydrophobic/nonpolar and hydrogen-bonding terms in predicting biological activity.

#### 2.1.2. Contours Maps Analysis (Field-Based)

**Figure 5:**
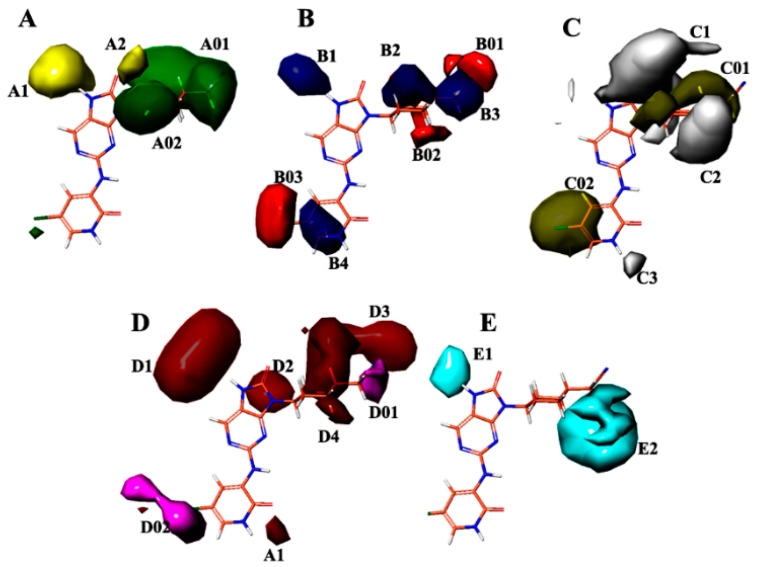
Field-based contour maps were employed in conjunction with compound 35 to elucidate the molecular effects. (**A**) Steric contour maps were generated, where green contours correspond to regions favoring steric interactions, while yellow contours represent regions disfavoring steric interactions. (**B**) Electrostatic contour maps were constructed, with blue contours signifying regions favoring positively charged substituents and red contours indicating regions favoring negatively charged substituents. (**C**) Hydrophobic contour maps were analyzed, with yellow contours denoting hydrophobic regions, which were favored, and grey contours indicating hydrophilic regions, which were disfavored. (**D**) Acceptor contour maps were examined, where magenta contours depicted regions favoring electron acceptor groups and red contours portray regions disfavoring electron acceptor groups. (**E**) H-bond donor contour maps were investigated, where cyan contours indicate regions favoring electron donor groups and purple contours reveal regions disfavoring electron donor groups.

In Figure 5A, contour maps depicting regions of favorable and unfavorable steric fields reveal that the inclusion of groups A01 and A02 elicits a favorable influence on enhancing biological activity, whereas the presence of groups A1 and A2 yields an unfavorable effect. Moving to Figure 5B, contour maps portraying regions of favorable and unfavorable electrostatic fields indicate that groups B1–B4 exhibit a favorable impact on promoting biological activity, whereas the introduction of groups B01–B03 results in an unfavorable effect. Turning our attention to Figure 5C, contour maps characterizing regions of favorable and unfavorable hydrophobic fields suggest that groups C01 and C02 contribute favorably to the enhancement of biological activity, while groups C1–C3 have an adverse effect. Proceeding to Figure 5D, contour maps displaying regions of favorable and unfavorable hydrogen-bond acceptor fields demonstrate that groups D1–D4 exert a favorable influence on increasing biological activity, whereas groups D01 and D02 have an unfavorable effect. Lastly, in Figure 5E, the contour map delineates the unfavorable region of the hydrogen-bond donor field, wherein the presence of groups E1–E2 exhibits an adverse effect on augmenting biological activity.

The contour maps presented in Figure 5A–E offer valuable insights into the impact of various molecular fields, including steric, electrostatic, hydrophobic and hydrogen bond acceptor/donor, on the modulation of biological activity. The findings provide evidence that specific molecular groups, namely A01, A02, B1–B4, A2, C01, C02 and D1–D5, exhibit favorable attributes in promoting biological activity. Conversely, the inclusion of groups A1–A2, B01–B03, C1–C3, D01 and D02 leads to an unfavorable effect on the enhancement in biological activity. These results have significant implications for the rational design of novel molecules with potential applications in biological or therapeutic settings.

#### 2.1.3. Contours Maps Analysis (Atom-Based)

**Figure 6:**
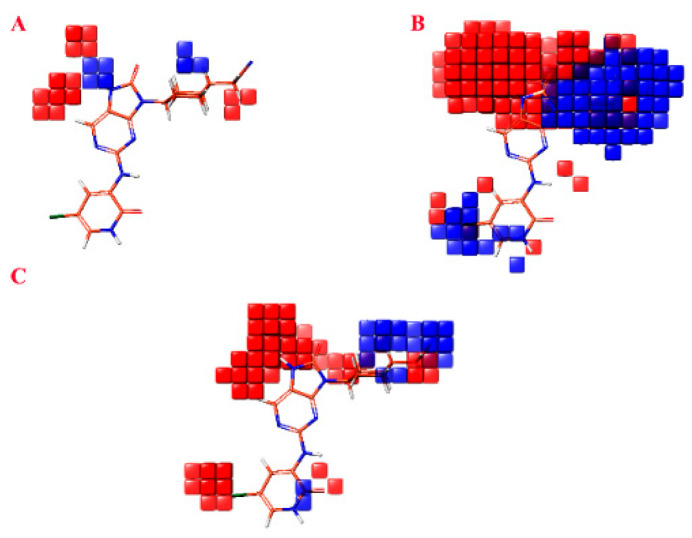
Atom-based contour maps where blue and red colors indicate favored and unfavored biological productivity, respectively. (**A**) H-bond donor. (**B**) Hydrophobic/non-polar. (**C**) Three withdrawing electrons.

The atom-based contour maps presented in Figure 6 illustrate the distribution of favorable (blue) and unfavorable (red) regions in terms of biological productivity. This analysis revealed that the red contours observed in Figure 6 correspond to an increase in biological productivity. Conversely, the red contours observed suggest an adverse impact on the enhancement in biological productivity.

In Figure 6A, the visualization of a blue cube proximal to the nitrogen atom and cyclohexane moiety signifies a region that acts as a hydrogen bond donor, thereby promoting biological activity. Conversely, the cubes situated on the bonded carbon or the nitrile moiety exhibit an unfavorable effect on the augmentation of biological activity. Moving to Figure 6B, the observation of a prominent red and blue cube on (9H-purin-9-yl) cyclohexyl) acetonitrile suggests that this specific region can either facilitate or impede biological activity due to the presence of non-polar hydrophobic groups. Additionally, the presence of a blue cube on 5-chloro-2-oxo-1,2-dihydropyridine indicates that non-polar hydrophobic groups within this region promote an increase in biological activity. Upon scrutinizing Figure 6C, it becomes apparent that the red cubes in proximity to 5-chloro-2-oxo-1,2-dihydropyridine and 5-chloropyridine impede the enhancement of biological activity. However, the presence of (cyclohexyl)acetonitrile near the blue cubes stimulates the increase in biological activity.

### 2.2. Pharmacophore Model

The results obtained from the pharmacophore analysis presented in Table 5 exhibit distinct values for each model. The survival score serves as an indicator of the overall performance of the model in predicting pharmacological properties, with higher scores indicating a superior model performance. The site score reflects the frequency of correspondence between the model and the known pharmacophore sites. The vector score signifies an optimal angle between different elements of the model, a crucial factor for prediction accuracy. The volume score represents the optimal size of the active cavity for effective interaction with the target molecule. The selectivity score quantifies the model’s ability to discriminate between active and inactive molecules. The num-matched score indicates the number of matches between the model elements and known pharmacophore sites. The inactive score measures the percentage of correctly identified inactive molecules by the model. The adjusted score is a modification of the survival score that considers the complexity of the model. The sites score represents the total count of corresponding pharmacophore sites identified by the model. Lastly, the PhaseHypo score evaluates the quality of the model’s hypothetical phase, which is crucial for accurate predictions.

**Figure 7:**
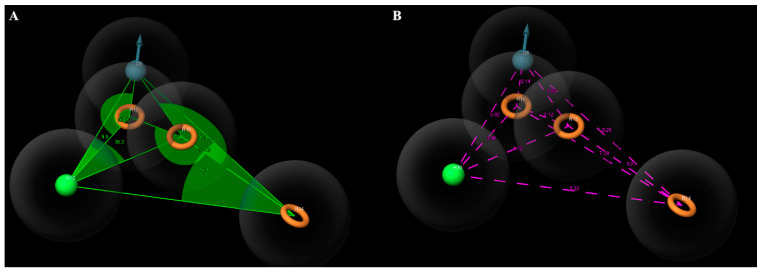
(**A**) Angles between the features of the DHRRR1 model. (**B**) Distances between the features of the DHRRR1 model.

The first model in the table demonstrates a relatively high survival score of 5.88, suggesting favorable predictive capabilities (Table 5 and Figure 7). Moreover, it exhibits a match count of 19, indicating proficiency in identifying known pharmacophore sites. The model also displays a selectivity value of 2.02, signifying its ability to discriminate between active and inactive molecules. With a volume value of 0.76, it appears to possess an optimally sized active cavity for effective interaction with the target molecule. The fit value of 3.61 takes into account the model’s complexity. Lastly, the hypothetical phase of the model was determined to be 8.55, indicating a high-quality phase that contributes to prediction accuracy. Overall, these results indicate that the first model holds promise as a suitable candidate for predicting pharmacological properties.

#### Comparing Field-Based and Atom-Based Models with the DHHHR Pharmacophore Model

The pharmacophore model predicts DHHHR1 for the biological activity against JAK3. It indicates the presence of three aromatic cycles, three hydrophobic features and one hydrogen-bond donor feature. On the other hand, the coefficients in the 3D-QSAR represent the relative importance of each factor (steric, electrostatic, hydrophobic, hydrogen-bond acceptor and hydrogen-bond donor) in predicting biological activity. In the DHHHR1 pharmacophore model, the hydrophobic features correspond to the most important coefficients in both models. In the field-based model, the hydrophobic factor has a coefficient of 0.206, suggesting its significant role in predicting biological activity. Furthermore, in the atom-based 3D-QSAR model, the hydrophobic/non-polar factor has a coefficient of 0.804, indicating its importance as well. The pharmacophore model also predicts the presence of a hydrogen-bond donor feature. In the field-based model, the hydrogen-bond donor factor has a coefficient of 0.029, while in the atom-based 3D-QSAR model, the hydrogen-bond donor factor also has a coefficient of 0.029. Although this coefficient is relatively low, it shows that this feature can contribute to the predicted biological activity.

The features in the pharmacophore model, such as the hydrophobic and hydrogen-bond donor features, correspond to the factors in the 3D-QSAR model with significant coefficients, suggesting a relationship between the two models in predicting the biological activity against JAK3.

### 2.3. Three-dimensional-QSAR Models Insights for Designing Novel JAK3 Ligands

**Figure 8:**
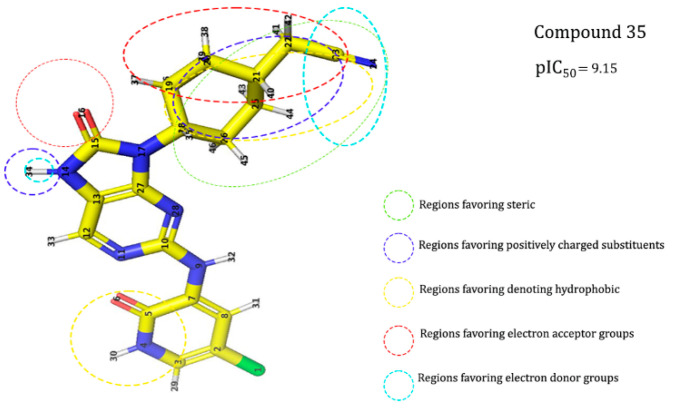
Mapping of the regions conducive to the development of biological activity against JAK3/STAT.

Figure 8 presents the essential information provided by the field-based and atom-based approaches for the design of novel ligands targeting anti_JAK3. The results of this analysis highlight the key molecular interactions between JAK3 and the ligands, with particular emphasis on the central role of Cy_s909_. These findings are of paramount importance for medicinal chemists and drug designers in the development of promising therapies aimed at targeting JAK3 and improving the treatment outcomes for patients with autoimmune diseases and cancer. The 3D-QSAR models’ results, summarized in Figure 8, lead us to designate the 13 JAK3 inhibitors, shown in Table 6 with their pIC_50_.

### 2.4. Pharmacophore Validation

The performance of prediction models is frequently assessed in molecular modeling using the H-validation technique. To train and test the model in a cross-validated fashion, this approach divides the dataset into several of groups, or subsets, and uses each of these groups.

Table 6 indicates that the pharmacophore model is effective. The model’s high true-positive rate and low false-positive rate are both shown by the ROC (receiver operating characteristic) value of 0.87. This shows that the model can properly discriminate between chemicals that are active and inactive. Other indicators of strong model performance are EF1 percent (1 percent enrichment factor) and BEDROC160.9 (BEDROC with an alpha value of 160.9). The model appears to be able to rank inactive compounds on average lower than active compounds, as seen by the average decoy ranking, which is also rather high at 4.29. This shows that the approach is successful at locating active substances and may be helpful in virtual screening initiatives.

**Figure 9:**
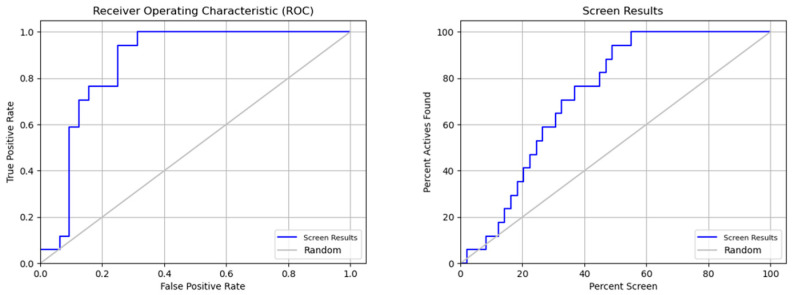
ROC analysis and screen results for the performance evaluation of screening.

The ROC curve as a function of the false positive rate (FPR) in Figure 9 indicates the performance of a binary classification model. The FPR represents the number of results incorrectly classified as positive compared to the total number of true negatives. The closer the ROC curve is to the upper left corner, the more the model is considered to be performing well. In this example, the model has a good performance because the ROC curve approaches the upper left corner, indicating that the model has a high sensitivity (ability to detect true positives) and low specificity (low false-positive rate).

### 2.5. Predicted Activity Using 3D-QSAR Models of New Ligands’ Design

The most optimal models for field-based and atom-based analyses were selected based on the findings presented in Table 7. Subsequently, these models were utilized to make predictions of the pIC_50_ values for the target molecules. The predicted pIC_50_ values were then compared to the experimental pIC_50_ values to evaluate the accuracy of the models. The results demonstrate that both the field-based and atom-based models exhibit robust predictive capabilities for the target molecules. The predicted pIC_50_ values closely align with the experimental values, with minimal absolute mean differences. These outcomes underscore the efficacy of both the field-based and atom-based models in forecasting the activity of molecules against the JAK3 target. Furthermore, these models can also be leveraged for the design of new molecules with anticipated activity against the JAK3 target, thereby significantly expediting the drug discovery process.

### 2.6. ADMET and Screening Using Covalent Docking

The ADMET study is a crucial step in the development of new drug compounds, enabling their pharmacokinetics and safety to be assessed [32]. 

The ADMET analysis of the new compounds predicted in Table 7 shows that compounds D2, D3, D11 and D12 exhibited the best LogS values (lower negative values under −4 indicate a lower solubility). D1, D5, D7 and D13 had optimal LogD (the logarithm of the distribution coefficient between octanol and water, indicating the lipophilicity of the compound) and LogP values (the logarithm of the partition coefficient between octanol and water, measuring the hydrophobicity of the compound). Compound D3 demonstrated the best intestinal absorption (HIA, indicating the percentage of the compound absorbed into the bloodstream after oral administration), whereas Caco-2 cell permeability was the highest for D13. MDCK permeability (measuring the compound’s ability to cross the blood–brain barrier) was optimal for D1 and D5, and D7 and D8 exhibited the best BBB (blood–brain barrier) penetration (indicating the ratio of the compound’s concentration in the brain to that in the blood). D2, D3 and D8 had the most suitable volumes of distribution (VDss, indicating the extent of the compound’s distribution in the body).

The inhibition of the CYP3A4 enzyme is crucial to JAK3 inhibition [33,34,35,36], as CYP3A4 is involved in the metabolism of many drugs, including JAK3 inhibitors. CYP3A4 belongs to the cytochrome P450 family. This family of enzymes is involved in the metabolism of drugs and other foreign substances (xenobiotics) in the body. By inhibiting CYP3A4, the plasma concentration of JAK3 inhibitors can be increased, enabling a more effective inhibition of JAK3 [37,38]. This may be particularly important for patients with autoimmune diseases, such as rheumatoid arthritis, where effective JAK3 inhibition can help to reduce inflammation and improve symptoms. Based on the results provided, it appears that all compounds are inhibitors of CYP3A4. D4 did not inhibit any metabolic enzyme, whereas D7 and D9 were the least inhibitory. D4, D9 and D10 were not metabolized, and clearance was good for all compounds. However, only D8 presented a risk of genotoxicity. 

The inhibition of JAK3 by the ligand can lead to a modification of the conformation of CYP3A4, which can disrupt its enzymatic activity. Several mechanisms can contribute to this inhibition [1,39]. Firstly, the modification of the conformation of CYP3A4 can alter the structure of its active site, which is responsible for substrate binding and drug metabolism. This can prevent substrate binding or alter catalysis by changing the spatial arrangement of active residues. Moreover, the modification of the conformation of CYP3A4 can disrupt the binding of NADPH, which is an essential cofactor for the enzymatic activity of CYP3A4. NADPH is required to provide electrons to CYP3A4, which are used to activate molecular oxygen and form reactive radicals that metabolize substrates [1,39,40,41]. Finally, the inhibition of JAK3 by the ligand can also affect the regulation of the expression of CYP3A4. Studies have shown that JAK3 signaling can regulate the expression of CYP3A4 by modifying the expression of nuclear receptors involved in its regulation. Therefore, the inhibition of JAK3 by the ligand can disrupt this regulation and affect the expression of CYP3A4. In conclusion, ligands with ADMET properties can inhibit the activity of CYP3A4 due to their ability to form a covalent bond with the Cys_909_ residue of JAK3. This interaction can disrupt the conformation of CYP3A4 and alter its enzymatic activity in several ways, which can have implications for drug metabolism and toxicity [42].

The results show that the newly designed compounds in Table 8 meet the drug-likeness criteria and have the potential to serve as promising leads for the development of new oral drugs. The compounds exhibited suitable physicochemical properties and complied with Lipinski’s rules for potential oral bioavailability.

### 2.7. Physicochemical Property

The results in Table 9 indicate that all compounds adhered to the established guidelines for the number of heavy atoms (HA, ranging from 8 to 12), the number of hydrogen-bond donors (HD, ranging from 3 to 7), the topological polar surface area (TPSA, ranging from 111.69 to 167.4 Å2), the number of rotations (nRot, ranging from 2 to 4), the number of rings (nRing, ranging from 4 to 5), the maximum number of rings (MaxRing, 9), the number of heteroatoms (nHet, ranging from 9 to 13), the hydrophilicity coefficient (fChar, ranging from 0 to 0), the number of stereogenic centers (nStereo, 0) and the molar mass (MW, ranging from 360.11 to 477.14 g/mol). Furthermore, all compounds complied with Lipinski’s rules for potential oral bioavailability, which include a molecular weight (MW) of less than 500, no more than five hydrogen-bond donors (HD ≤ 5), no more than ten heavy atoms (HA ≤ 10) and a LogP value lower than or equal to 5. In conclusion, the series of 13 polycyclic organic compounds meet the drug-likeness criteria and have the potential to serve as promising leads for the development of new oral drugs. 

The compounds D1, D2 and D3 share a common core structure of 2-amino-7,9-dihydro-8H-purin-8-one, with only substitutions in the aromatic ring differing between them. Specifically, D1 contains a pyridine ring, D2 includes a phenyl ring with a pyridine moiety, while D3 has a phenyl ring. The results of covalent docking allowed us to evaluate the affinity (Kcal/mol) of D1 for the newly designed groups using 3D-QSAR and pharmacophore models. The compound 5-chloro-6-(1H-imidazol-4-yl)pyridin-2(1H)-one interacts with several residues, including a and c, as well as d and g, which correspond to the group 2-(piperidin-1-yl)acetonitrile. Notably, D1 exhibited good ADMET properties and acted as a CYPA34 inhibitor. Compared to tofacitinib, the new predicted molecules exhibited good ADMET and physicochemical properties.

The utilization of computer modeling in chemistry simplifies and guides the design of new groups for each molecule with advantageous pharmacokinetic and ADMET features. Moreover, the models employed in this study can serve as a point of reference for future research.

### 2.8. Covalent Docking (CovDock)

Research based on the freshly discovered crystal structure of JAK3 (PDB ID: 4Z16), for example, demonstrated that CovDock could properly predict the binding modes and affinities of newly developed compounds that establish a covalent bond with the Cys_909_ residue in JAK3 [3]. This approach has demonstrated a good resolution and better ligand structure goodness of fit to experimental data. On the other hand, molecular docking has been used to study the interaction between tofacitinib and JAK3 [43,44]. Tofacitinib is known to have solubility issues, but molecular docking has been employed to predict its binding mode and identify potential modifications that could improve its solubility and efficacy.

**Figure 10:**
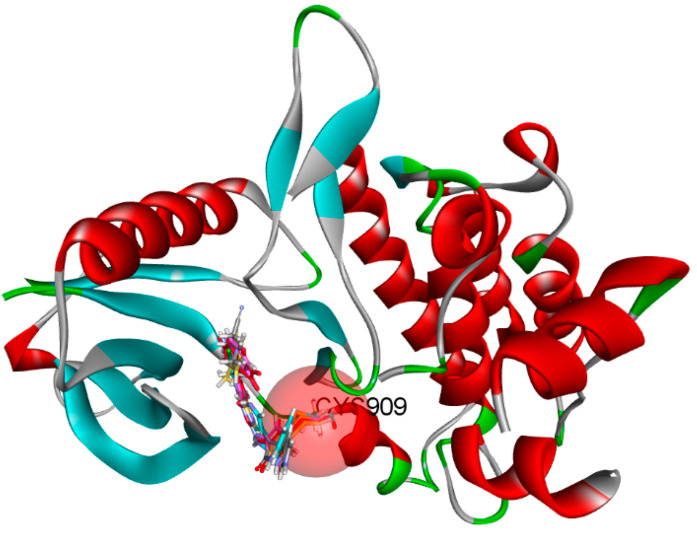
Covalent bonds with Cys_909_ in protein interaction pocket.

By combining the ADMET study with a CovDock study, molecules can be filtered to select only those with a high affinity for the therapeutic target [45]. Indeed, drugs need to interact specifically and strongly with their biological target if they are to be effective. Following ADMET analysis, the molecules selected for CovDock, the first five compounds with high affinity, were chosen for further study, as shown in Figure 10 and Table 10, and were able to form a covalent bond with residue Cys_909_. 

**Figure 11:**
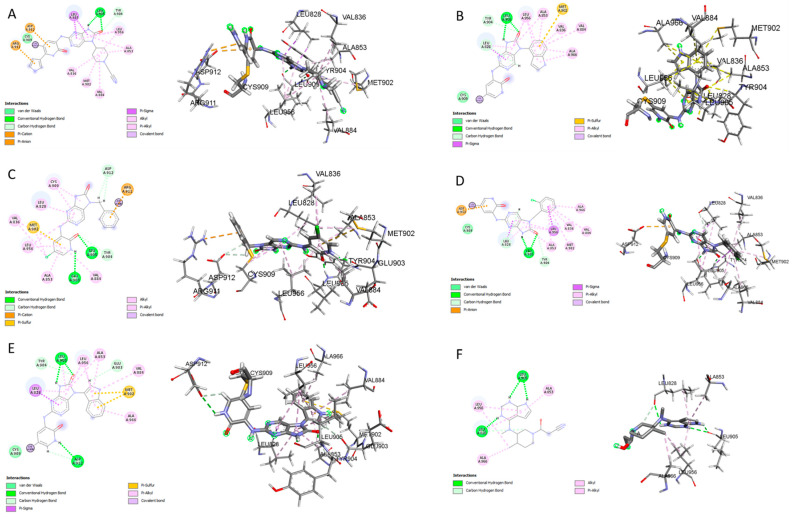
Two-dimensional and three-dimensional interactions of the newly designed compounds with JAK3 protein using CovDock analysis. Figure shows the docking analyses for the new compounds, D1 to D5, which favor (**A**–**E**), respectively. (**F**) favors Tofacitinib, which is FDA-approved.

The CovDock analysis revealed that compound D1 interacts with JAK3 protein residues in several ways (Figure 11A). Specifically, it forms two hydrogen bonds with residue LEU905, a carbon–hydrogen bond with TYR904, a Pi–cation bond with ARG911, a Pi–anion bond with ASP912, as well as two Pi–sigma bonds with LEU828. Additionally, it interacts with VAL836, ALA853, VAL884, MET902 and LEU956 through alkyl-type interactions. Finally, Pi–alkyl bonds are observed with residues ALA853, LEU956 and an unidentified molecule. These findings suggest that compound D1 has potential as a JAK3 protein inhibitor. 

Similarly, the CovDock analysis of D2 indicated that this compound interacts with JAK3 protein residues through several bonds (Figure 11B). It forms two hydrogen bonds with LEU905, a carbon–hydrogen bond with TYR904, and another with LEU828, as well as a Pi–sigma interaction with LEU828 and a Pi–sulfide bond with MET902. In addition, it interacts with LEU956, LEU828, ALA853, VAL836, ALA853, VAL884, LEU956, ALA966 and LEU956 through alkyl-type interactions, at varying distances. These interactions suggest that D2 also has potential as a JAK3 inhibitor. D3 (Figure 11C) indicates that this compound interacts with JAK3 protein residues through two hydrogen bonds with LEU905 and GLU903, a carbon–hydrogen bond with TYR904, two carbon–hydrogen bonds with ASP912, a Pi–cation bond with ARG911, and a Pi–sulfide bond with MET902. It also interacts with VAL836, MET902, VAL836, ALA853, VAL884, LEU956, LEU828 and CYS_909_ through Pi–alkyl type interactions, at varying distances. These varied interactions suggest that D3 could be a potential JAK3 protein inhibitor. D4 (Figure 11D) indicates that this compound interacts with JAK3 protein residues through two hydrogen bonds with LEU905, a carbon–hydrogen bond with TYR904, another with LEU828, a Pi–anion interaction with ASP912, and two Pi–sigma bonds with LEU828 and LEU956. It also interacts with LEU956, LEU828, ALA853, VAL836, ALA853, VAL884, MET902 and ALA966 through alkyl-type interactions, at varying distances. These interactions suggest that D4 also has potential as a JAK3 protein inhibitor. D5 (Figure 11E) indicates that this compound interacts with JAK3 protein residues through three hydrogen bonds with LEU905 and ASP912, a carbon–hydrogen bond with TYR904, and two carbon–hydrogen bonds with ASP912 and GLU903. It also forms Pi–sigma interactions with LEU828 and Pi–sulfide bonds with MET902. Additionally, D5 interacts with LEU956, LEU828, ALA853, LEU905, VAL884, LEU956, ALA853, LEU956, LEU956 and ALA966 through alkyl-type interactions, at varying distances. These varied interactions suggest that D5 could be a potential inhibitor of the JAK3 protein. Finally, at distances of 3.04, 2.75, 2.08 and 3.78, tofacitinib established numerous hydrogen bonds with residues Leu828 and Leu906. Leu956, Ala966, Leu828 (4.35), Ala853 (4.56) and Leu956 (4.39) were among the residues with which tofacitinib interacted hydrophobically (Figure 11F).

The results generated by the CovDock analysis of compounds D1 to D5 suggest that all these compounds have potential as JAK3 inhibitors. The affinity values obtained by CovDock for these compounds range from −7.37 to −9.55 Kcal/mol, indicating that they have a good binding affinity with the JAK3 protein (Table 10). In conclusion, the presence of a specific cysteine residue, Cys_909_, in the enzyme’s active site makes CovDock particularly relevant for the discovery of JAK3 inhibitors. 

The D1–D5 compounds analyzed in this study were able to form a covalent bond with Cys_909_ and interacted with several JAK3 protein residues through multiple types of bonds and interactions, suggesting their potential as JAK3 protein inhibitors. The study highlights the importance of CovDock in drug discovery and the potential for the development of new drugs for the treatment of autoimmune diseases, such as rheumatoid arthritis. The hydrogen bonds observed between the molecules and the protein residues, as well as the other types of interactions, such as Pi–cation, Pi–anion and Pi–sigma bonds, indicate the importance of hydrogen bonding in the molecular recognition process, which can be exploited in the design of new drugs with improved affinity and selectivity.

### 2.9. Molecular Dynamics Simulation Analysis

**Figure 12:**
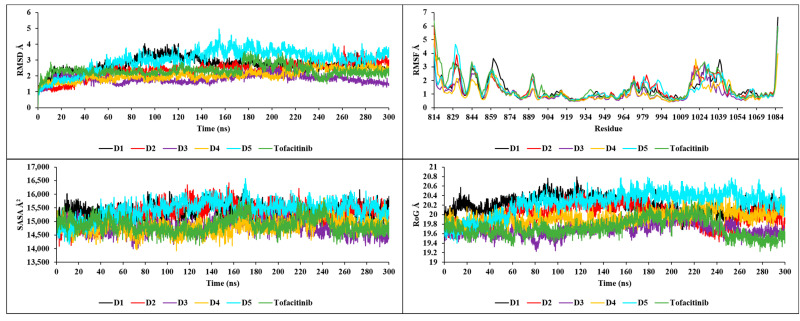
RMSD, RMSF, SASA and RoG plots of the newly designed compounds.

The RMSD results shown in Figure 12 illustrate the high degree of stability of the new compounds in interaction with JAK3. Compound D1 demonstrated stability with an RMSD between 2.5 and 3 Å, compound D2 with an RMSD between 2 and 2.5 Å, compound D3 with an RMSD between 1.5 and 2 Å, compound D4 with an RMSD between 1 and 2.5 Å and finally, compound D5 with an RMSD between 2.5 and 3.5 Å. The analysis of the graphs shows that the most stable RMSDs follow the following order: D3, D2, D4, D1 and finally D5. The RMSF analysis shows a similar stability for the residues of the complexes formed by the new compounds with JAK3 (Figure 12), except the common residues showing an RMSF greater than 3 Å (ARG1086, PRO814, ARG1085, GLY861, ILE1040, PRO862, ASN1028, ASP863, GLY831, ILE1041, ASN832, LYS830, ILE1039, SER860, PHE833, GLN864, PRO814, ARG1086, THR815, ARG1085, ASN832, ILE816, GLY831, PHE833, LEU844, ASN1022, ASN1020, ASN1028, ARG1084, ASN1021, GLY834, LYS830, ASN1031, ASP846, ASN1030 and GLY845), which lie outside the pocket site of the JAK3 active site. Both SASA and RoG calculation results indicate that all compounds retain their structure during the 300 ns simulation (Figure 12). During the simulation, an average SASA of 15,500 Å^2^ was observed for D1, D2 and D5, and 14570 Å^2^ for D3, D4 and tofacitinib, with remarkable stability. The same was observed for RoG, with an average of 20.2 Å for D1, D2 and D5, and 19.6 Å for D3, D4 and tofacitinib. This means that the compactable structures can maintain their shape and surface area even in the presence of a solvent. Additionally, the stable RoG values indicate that the structures of the complexes are able to maintain their compactness and shape during the simulation.

**Figure 13:**
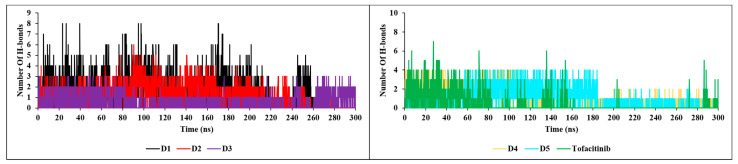
H-bonds plots of the newly designed compounds.

The nH-bond analysis in Figure 13 shows that the newly designed compounds exhibit stability with a minimum number of one bond and a maximum number of up to nine bonds during the 300 ns simulation, with no extraneous occupancies. These results confirm previous findings for RMSD, RMSF, SASA and RoG, which also testify to the stability of complexes formed with JAK3. The new compounds show a remarkable affinity for JAK3, with high structural stability during the 300 ns simulation. 

#### 2.9.1. DSSP Analysis

**Figure 14:**
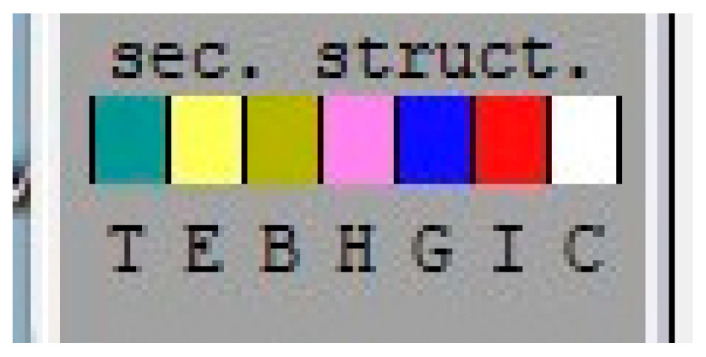
Codes used in the DSSP program.

The letters T, E, B, H, G, I and C are codes used in the DSSP (Dictionary of Secondary Structure of Proteins) program to describe the secondary structure of proteins (Figure 14). Their meanings are: T: turn; E: extended strand in parallel and/or anti-parallel β-sheet conformation; B: residue in isolated β-bridge; H: α-helix; G: 3-helix; I: 5-helix; and C: coil (coiled or unstructured structure). These codes describe the local conformation of each protein residue in terms of its helical, β-sheet or loop conformation. The DSSP program is very useful for analyzing protein structure and predicting function.

**Figure 15:**
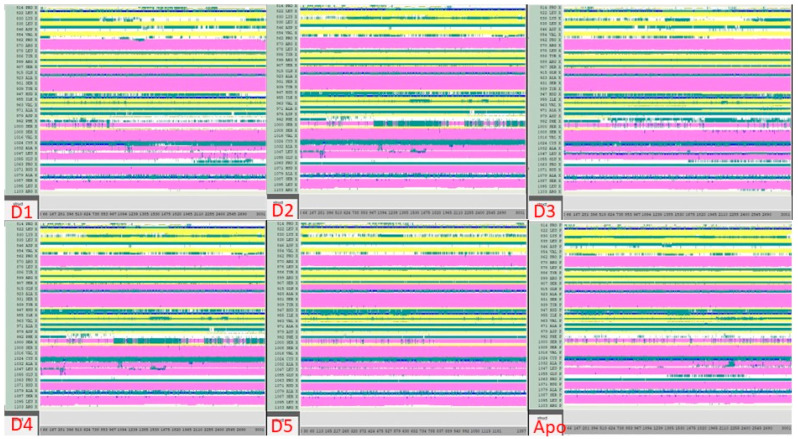
Analysis of the newly designed compounds compared to apoprotein using DSSP.

The DSSP analysis shows that the complexes formed between compounds D1–D5 and JAK3 (Figure 15), compared to the apoprotein, exhibit similar structures with some exceptions for certain transitions between the T, E, B, H, G and I conformations. Overall, however, the comparisons show a strong similarity between the complexes formed, suggesting that the complexes tend to be stable. This may be due to the nature of the interaction between the compounds and JAK3, which allows a stable conformation of the complexes formed. This analysis is important because it suggests that the D1–D5 compounds have the ability to form stable complexes with JAK3, which may be beneficial for the development of drugs for the treatment of diseases linked to JAK3 activity. In conclusion, the results of the DSSP analysis suggest that the complexes formed between compounds D1–D5 and JAK3 are stable and that these compounds can be considered potential candidates for the development of drugs targeting JAK3. 

#### 2.9.2. Free Energy Landscape Analysis (FEL)

**Figure 16:**
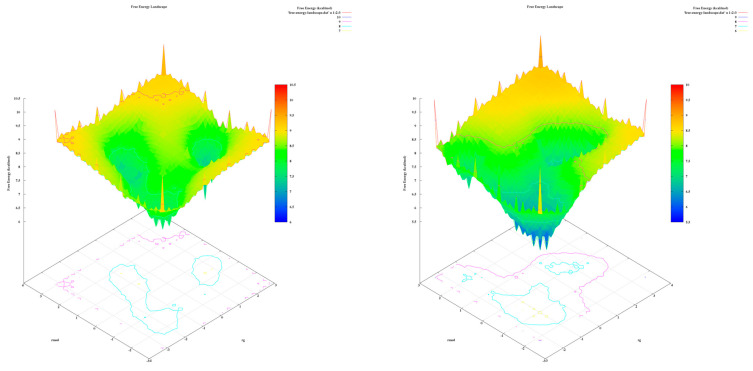
Free energy landscape of compounds D1 and D2.

**Figure 17:**
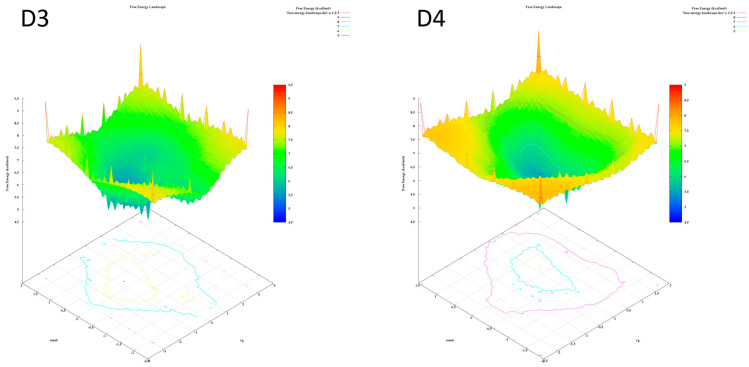
Free energy landscape of compounds D3 and D4.

**Figure 18:**
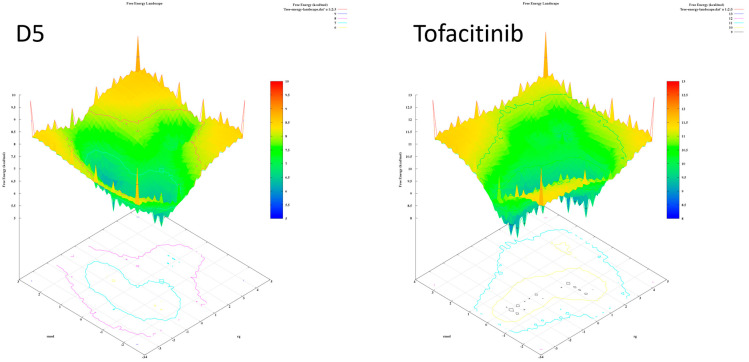
Free energy landscape of compounds D5 and tofacitinib.

**Figure 19:**
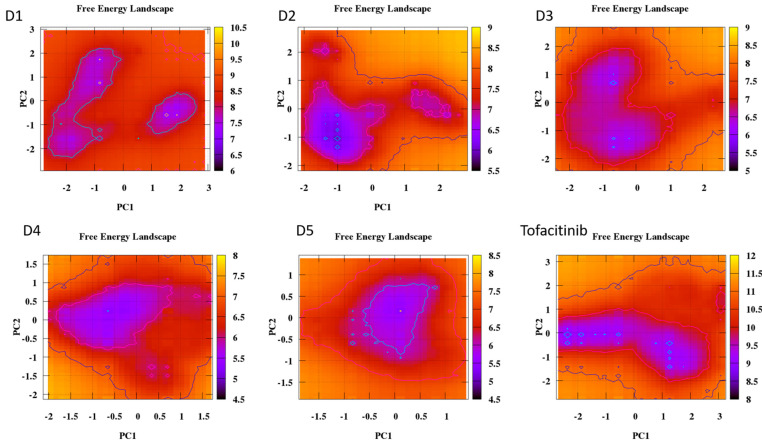
Free energy landscape of all compounds with Tofacitinib drug.

The analysis of the FEL for the design of new compounds includes folding spots along the PC2 and PC1. D1 and D2 represent two folding spots located between −2.3 and −2.2, with average free energy landscape energies of 7.7 and 7 kcal/mol, respectively (Figure 16. Compounds D3 and D4 show a single folding spot located between −2.1 and −1.5, with an average free energy landscape of 6.5 kcal/mol for both (Figure 17). D5 represents a single folding spot located between −2 and 2, with an average free energy landscape of 7 kcal/mol (Figure 18 and Figure 19). As for tofacitinib, it represents a single folding spot located between the PC2 and PC1 of −2 and 3, with an average free energy landscape of 9 kcal/mol (Figure 18 and Figure 19).

According to the analysis of the results, compounds with a single folding spot indicate that the structure has not undergone additional folding changes (complexes), but overall, the structures demonstrate stability based on the landscape free energy. Finally, the comparison between the new ligands and FDA-approved tofacitinib indicates that the new inhibitors exhibit less stability than tofacitinib (low energy implies reduced stability).

### 2.10. MM/GBSA Analysis

In drug discovery, the MM/GBSA method is widely employed to assess potential drug candidates and optimize their binding affinity. The outcomes of the MM/GBSA analysis offer valuable insights into the pivotal residues implicated in the binding interaction, thereby guiding the rational design of novel compounds with enhanced binding affinity. The results presented in Table 11 show the MM/GBSA outcomes during the 300 ns simulation for the new compounds predicted with tofacitinib.

The MMGBSA method was used to estimate binding free energy from molecular dynamics simulations. Table 11 shows that the main contributions to binding energy come from van der Waals interactions (Δ_VDWAALS_), electrostatic interactions (ΔE_EL_), energy due to cavitation/solvation (ΔE_GB_) and surface interactions (ΔE_SURF_). The values of ΔV_DWAALS_ and ΔE_EL_ for the new compounds (D1–D5) were all more negative than those of tofacitinib. This indicates that the new compounds have stronger van der Waals and electrostatic interactions with the target protein compared to tofacitinib, which could potentially lead to more stable binding. The values of ΔE_GB_ for all the compounds (D1–D5) were positive, indicating that energy is required for cavitation and solvation. However, the values are lower than that of tofacitinib, suggesting that the new compounds may have less solvation energy, which could contribute to their favorable binding. The values of ΔE_SURF_ for all the compounds (D1–D5) and tofacitinib were negative, indicating favorable surface interactions between the ligands and the target protein. The values of ΔG_GAS_ for all the compounds (D1–D5) and tofacitinib were negative, indicating that the gas-phase energy of the complexes is favorable. However, the values for D3 and D4 were more negative than those of the other compounds, suggesting that they have stronger gas-phase interactions, potentially contributing to their stability. The values of ΔG_SOLV_ for all the compounds (D1–D5) were positive, indicating that energy is required for solvation. However, the values were lower than that of tofacitinib, suggesting that the new compounds may have less solvation energy, which could be advantageous for binding. The values of Δ_TOTAL_ for all the compounds (D1–D5) were more negative than that of tofacitinib. This indicates that the new compounds, on average, have more favorable binding energies compared to tofacitinib, suggesting their potential as better JAK3 inhibitors. The comparative analysis of the Δ_Energy_ values shows that the new compounds (D1–D5) generally exhibit more favorable interactions with the target protein compared to tofacitinib. D3 stands out with the most negative Δ_TOTAL_ value, indicating that it could potentially form the most thermodynamically stable complex among the studied compounds. 

## 3. Conclusions

In this study, novel medications for RA, one of the most common autoimmune illnesses globally, were sought using molecular modeling approaches. The search for JAK3 inhibitors, crucial enzymes for treating autoimmune illnesses, such as RA, was the main objective of the study. The 3D-QSAR approach was employed to predict new JAK3 inhibitors, and two robust models were developed, yielding good results by identifying groups that can readily direct their interaction. A reliable pharmacophore model was provided to enable the clear characterization of chemical features, leading to the design of 13 inhibitors with their pIC_50_. CovDock was used to investigate the binding affinity of these inhibitors, revealing a strong affinity. Among the 13 inhibitors, 5 showed a high affinity, greater than −8 kcal/mol. To confirm these results, MD simulations and free energy binding calculations were performed for 300 ns. The results of these analyses suggest that the newly predicted molecules have promising potential as JAK3 inhibitors for the treatment of rheumatoid arthritis.

In conclusion, this study provides valuable insights into the development of new drugs for the treatment of RA. The researchers’ use of molecular modeling techniques allowed them to identify promising JAK3 inhibitors with favorable strong binding affinity and favorable stability. The results of this study provide a foundation for further research and the development of these inhibitors as potential drugs for the treatment of RA.

## 4. Methods and Materials

### 4.1. Data Set

The dataset employed in this study comprised a series of 35 molecules (Table 12) with reported IC_50_ (*Exp*) values against the JAK3 target, as indicated by previous investigations [16]. Such datasets are widely employed in pharmacological studies to evaluate the efficacy of molecules targeting JAK3, a pivotal player in numerous physiological processes, including immune response regulation. To facilitate analysis, the IC_50_ values were converted to pIC_50_, a logarithmic measure reflecting the activity of the molecules. Within this dataset, 28 molecules were allocated for training purposes, while the remaining 7 molecules were designated for testing. The molecule exhibiting the highest activity within the training set was identified and utilized as a reference to gauge the activity of other molecules. The partitioning of the dataset into training and test sets was performed randomly to ensure an equitable distribution of data. It is noteworthy that this dataset has not yet undergone computational modeling processes. Nevertheless, it provides a robust foundation for subsequent analysis and modeling endeavors, which hold potential for the discovery of novel compounds exhibiting efficacy against the JAK3 target. Through the examination of pIC_50_ values within the training set, researchers can identify shared characteristics among active molecules, thereby facilitating the design or prediction of novel molecules with enhanced activity.

#### Software

In the present investigation, a comprehensive array of computational methodologies was employed to identify potential ligands exhibiting enhanced activity towards a specific target [30,46]. Specifically, the Schrödinger software 2021 suite was utilized to execute an analysis, CovDock studies, and the formulation of a pharmacophore hypothesis [47]. Molecular dynamics (MD) simulations and MM/GBSA calculations were performed using the latest version of the GROMACS software (2021) [48,49]. The Swiss ADME and ADMETlab 2.0 web servers were leveraged to evaluate the ADMET properties of the identified compounds [50,51,52]. The three-dimensional structures of the ligands were visualized using BIOVIA Discovery Studio [53], and the design of new molecules was facilitated using ChemSketch [54].

### 4.2. Three-Dimensional-QSAR

Field-based methodology relies on the characterization of electrostatic, van der Waals and steric fields to elucidate molecular interactions. This approach is widely employed to discern the intricate interactions occurring between a given molecule and its biological target [55,56]. By calculating these fields in the vicinity of the molecule, a predictive model can be constructed to estimate the biological activity of the compound. Conversely, the atom-based method relies on atomic descriptors to depict the molecular structure [57]. Atomic descriptors are derived from various atom-specific properties, including size and electronegativity. Both approaches, field-based and atom-based, provide valuable information on the groups that favor and disfavor the increase in biological activity, leading to the easier design of new compounds and predicting their activity.

### 4.3. QSAR Methodology

The 35 structures encompassed in the dataset were meticulously aligned through the utilization of Schrödinger software 2021 [47]. To create distinct training and test sets, a random splitting method was employed, with an approximate ratio of 80:20 (28 compounds allocated to the training set and 7 compounds assigned to the test set). During the training process of the models, the OPLS_2005 force field was implemented [58]. The calculation of the fields was conducted on an orthorhombic grid, with a spacing of 1 Å, extending 3 Å beyond the boundaries of the training set molecules. For van der Waals and electrostatic interactions, a threshold of 30 kcal/mol was set, while any points situated within a distance of 2 Å from any of the atoms in the training set were eliminated. In the subsequent PLS (partial least squares) procedure, variables with a standard deviation below 0.01 were excluded, and variables exhibiting regression coefficients highly sensitive to minor alterations in the composition of the training set were eliminated by employing a |*t*-value| < 2.00 filter [59,60,61,62]. The maximum number of PLS factors was defined as 1. An atom-based model was developed for each group of compounds sharing identical or similar scaffolds [63]. The atoms were categorized into specific atom types, such as hydrogen-bond donor (D), hydrophobic or nonpolar (H), negative ionic (N), positive ionic (P), electron-withdrawing (including hydrogen-bond acceptors, W), and miscellaneous (X) [64,65]. Cubes with a 1 Å grid were defined to encompass the space occupied by the aligned conformations, and each cube was assigned a value of zero or one depending on the presence or absence of atoms or sites. The molecules were represented by binary strings of zeros and ones, which functioned as independent variables. The QSAR models were established by incorporating PLS factors into these independent variables, with a set of four PLS factors to prevent overfitting. Regression coefficients were assigned to each bit to identify specific chemical features that positively or negatively influenced the activity. The predictive ability of the QSAR model was evaluated through a leave-one-out (LOO) cross-validation analysis. Consequently, a series of models were generated [66].

### 4.4. Pharmacophore Hypothesis

The pharmacophore hypothesis, a widely adopted approach in pharmaceutical chemistry, serves to identify and model the crucial interactions between a drug molecule and its biological target. This approach is rooted in the understanding that specific structural or chemical attributes of the molecule play a pivotal role in its biological activity [67,68]. The sophisticated Schrödinger software version 2021 offers advanced tools for generating and validating pharmacophore hypotheses by leveraging information of molecular interactions, including hydrogen bonds, electrostatic interactions and hydrophobic interactions [69].

**Figure 20:**
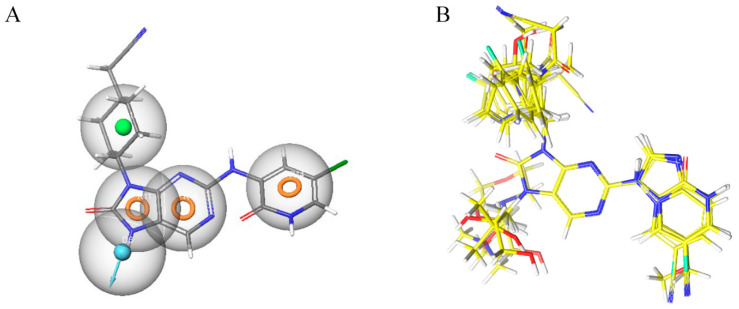
(**A**) Illustration of the significant model features associated with the reference molecule exhibiting the highest pIC_50_ values (pIC_50_ = 9.15). (**B**) Alignment of molecules based on the pharmacophore model.

To prepare the structure data file for our test compounds, we employed the LigPrep panel integrated within the Schrödinger software version 2021 [47]. Ligand chemistry was appropriately normalized and extrapolated for pharmacophore modeling using PHASE, an automated process that aligns the ligands based on their optimal arrangement and shared properties (Figure 20). Subsequently, the prepared ligands were imported into the Maestro workspace, and their experimental binding affinities (pIC_50_) were utilized to categorize them as active or inactive, with pIC_50_ values derived from the equation pIC_50_ = −log (IC_50_). An IC_50_ affinity of ≤ 50 nM corresponded to a pIC_50_ value exceeding 6.0, while a threshold of 10 µM or a pIC_50_ value below 7.0 was employed to identify inactive molecules. The assumption requirement was set to match at least 50% of the active compounds, and a minimum of five features were preferred for a successful match. The assumption difference criteria remained at their default settings, except for donor and negative molecules, where ionic features were assigned a value of 1 to ensure compatibility between the acceptor and negative features.

### 4.5. ADMET

The suboptimal pharmacokinetic characteristics of lead-like molecules constitute a pivotal contributor to the high rate of clinical trial failures. To mitigate this challenge, and concurrently economize time and resources, in silico assessment can be leveraged during the early stages of drug design and development, before the progression of lead-like molecules into preclinical studies [26,27,34,70].

### 4.6. Molecular Docking (MD)

Molecular dynamics simulations are a valuable tool for investigating a diverse range of phenomena, encompassing aspects such as protein stability and flexibility, receptor–ligand interactions and the dynamics of water molecules surrounding biomolecules. 

Standard molecular docking and CovDock are two different techniques used to study the interactions between a ligand and a protein. Standard molecular docking relies on the use of algorithms to predict the most favorable conformation of the ligand–protein complex, based on binding energy [71]. Covalent molecular docking, on the other hand, takes into account covalent interactions between the ligand and the protein. In this case, the ligand is designed to contain a reactive function that can form a covalent bond with a specific residue of the protein. This method is particularly useful for studying enzymes and proteins involved in diseases, as it allows for specific targeting of the active residues of the protein [72].

In the present study, several key metrics were calculated for both systems [34,45], including root-mean-square deviation (RMSD), root-mean-square fluctuation (RMSF), solvent-accessible surface area (SASA), radius of gyration (RoG) and hydrogen-bond analysis (H-bond). RMSD measures the deviation of atomic positions, revealing stability and conformational changes over time. It helps to assess a molecular model’s accuracy and compare different structures. RMSF quantifies atom or residue fluctuations, identifying flexible regions related to dynamics and ligand binding sites. SASA calculates the accessible surface area, shedding light on protein–water interactions and buried regions. It is useful for studying folding, protein–protein interactions and ligand binding. RoG characterizes overall compactness and shape, providing information on global conformational properties and transitions. H-bond analysis examines hydrogen bond formation and stability, crucial for molecular recognition and binding specificity.

#### 4.6.1. CovDock and Molecular Docking-Based Virtual Screening

Molecular docking investigations were executed employing the Schrodinger Maestro software version 2021. Specifically, the X-ray structures of Tyrosine kinase 3 with the Protein Data Bank identification code 4Z16, characterized by a resolution of 2.90 Å, were chosen as the docking targets for the CovDock simulations [30]. Before docking, the protein structure underwent preparatory procedures using Maestro’s Protein Preparation Wizard. These included the addition of hydrogen atoms, removal of water molecules and adjustment of tautomeric and protonation states of the amino acids. Subsequently, the crystal structure’s energy was minimized by utilizing the OPLS2005 force field. As for the ligands, they were prepared using the LigPrep module in Schrodinger. Ionizer was employed to generate ligand structures at a physiological pH of 7.4, followed by energy minimization using the OPLS2005 force field. In preparation for molecular docking, the reactive amino acid residue Cys_909_ was temporarily mutated to a residue more amenable to accommodating the pre-reactive conformations of the ligands. The receptor grid for docking was generated, with the reactive functional groups of the ligands constrained within a 5 Å vicinity of the Cβ atom of the reactive amino acid residue, employing the standard Glide XP mode. Post-docking minimization was performed, and up to three optimal-fit poses were recorded for each ligand. All poses were subjected to meticulous manual inspection, and the pose with the most favorable docking score was chosen, unless otherwise specified. The amino acid residue was then reverted back to Cy_s909_, and conformational sampling was performed using Prime VSGB2.0 coupled with the OPLS2005 force field. CovDock simulations were conducted utilizing the CovDock application, with Cys_909_ serving as the nucleophilic residue. This nucleophilic residue underwent conjugate addition to the predefined carbonyl activation site within CovDock. The formation of the covalent bond was ascertained for ligand poses featuring reactive functional groups within a 5 Å range, following the specified reaction scheme. Ligand selection and ranking were based on the Glide scores of the binding modes observed in the pre-reactive complexes.

#### 4.6.2. Molecular Docking Standard (MDS)

Before molecular docking, we optimized the ligands to be docked using Avogadro software 2.0. Next, we downloaded the structure of JAK3 from the RCSB database (PDB ID: 4Z16). The 4Z16 crystal complex consists of water molecules and the co-crystallized ligand 4LH with the protein 4Z16. We prepared the protein by removing all water molecules and the co-crystallized ligand from the protein and adding polar hydrogens to the JAK3 protein structure using Discovery Studiosoftware 2021. The active site of 4Z16 is defined by the sphere containing the co-crystallized ligand (4LH). Once the ligand and protein were prepared, we performed molecular docking using AD4 and AutoVina. The three-dimensional grid was defined using the AUTOGRID algorithm, which determines the binding energy of ligands with their receptor [73]. The default grid size is x = 60, y = 60, and z = 60, with a distance between grid points of 0.375 Å [73]. The center of the grid is the active site of the receptor with coordinates (x = −6.68875 Å, y = −14.7757 Å, and z = 1.89597 Å). The docking results obtained by AD4 and Vina were visualized using Discovery Studio software 2021. CovDock, the guide for the procedure, explains the several actions that may be taken to complete the CovDock approach utilizing AD4 with flexible side chains [74].

### 4.7. MD Simulation

Molecular dynamics (MD) simulations were performed employing the GROMACS MD engine. The input files for the simulations were generated using CHARMM-GUI [75], utilizing the CHARMM36 force field for system calculation [76]. The system was solvated in a cubic box using the TIP3P water model, with a padding of 10 Å. To ensure system neutrality, NaCl salt was added at an ionic concentration of 0.15 M, and the Monte Carlo method was employed for ion positioning [77]. A gradient descent method was applied to minimize the energy of the system over a duration of 10,000 steps. Following the energy minimization, the system was equilibrated in a constant atom number, volume and temperature (NVT) ensemble at a temperature of 310 K for 30 ns. Subsequently, the system underwent unrestricted MD simulations for a duration of 300 ns in a constant number of atoms, pressure and temperature (NPT) ensemble, with a reference temperature of 310 K and pressure of 1 atm. Trajectory MD analyses were conducted using the Visual Molecular Dynamics (VMD) software 2020 to examine system stability and generate essential parameters, such as root-mean-square deviation (RMSD), root-mean-square fluctuation (RMSF), radius of gyration (RoG), protein solvent accessible surface area (SASA) and H-bond analysis [34,78,79].

### 4.8. Free Binding Energy (MM/GBSA)

The assessment of the binding affinity between receptors and small ligands can be determined by analyzing the binding free energy. In this particular study, the molecular mechanics/generalized Born surface area (MM/GBSA) method was used to calculate the binding free energy using the AMBER 14 software to calculate the binding free energy [49,80]. The calculation of Equations (1)–(7) used in this study are presented below [27]: Δ_VDWAALS_, ΔE_EL_, ΔE_GB_, ΔE_SURF_, ΔG_GAS_, ΔG_SOLV_ and Δ_TOTAL_. 

Δ_VDWAALS_: This term represents the van der Waals interaction energy between the protein–ligand complex and its surroundings [81].

(1) $$\Delta_{\mathrm{VDWAALS}}\;=\sum^{i}\sum^{j}6\varepsilon \left[{\left(\frac{\sigma_{ij}}{r_{ij}}\right)}^{12}-2{\left(\frac{\sigma_{ij}}{r_{ij}}\right)}^{6}\right],$$

where ε is the energy scaling factor, σ*_ij_* is the distance at which the potential energy of the interaction between atoms *i* and *j* is zero, *r_ij_* is the distance between atoms *i* and *j*, and the summations are over all pairs of atoms *i* and *j*. The equation is based on the Lennard–Jones potential.

ΔE_EL_: This term represents the electrostatic interaction energy between the protein–ligand complex and its surroundings [82,83].

(2) $$\Delta E_{\mathrm{EL}}=\sum^{i}\sum^{j}\frac{q_{i}*q_{j}}{\varepsilon r}$$

where *q_i_* and *q_j_* are the partial charges on atoms *i* and *j*, r is the distance between atoms *i* and *j*, ε is the dielectric constant of the solvent, and the summations are over all pairs of atoms *i* and *j*. The equation is based on Coulomb’s law.

ΔE_GB_: This term represents the energy change associated with the molecule’s transfer from a vacuum to a solution [84].

(3) $$\Delta E_{\mathrm{GB}}=\gamma \sum^{i}\frac{q_{i}{}^{2}}{r_{i}}+k\sum^{i}\sum^{j}\frac{q_{i}*q_{j}}{r_{ij}}+\;\sum^{i}\sigma_{i},$$

where γ and *κ* are constants that depend on the solvent dielectric constant and ionic strength, *q_i_* is the partial charge on atom *i*, *r_i_* is the distance from atom *i* to the center of the solvent-accessible surface, *r_ij_* is the distance between atoms *i* and *j*, and σ*_i_* is a surface tension term that penalizes the creation of a solvent-accessible surface. The equation is based on the generalized Born model.

ΔE_SURF_: This term represents the energy change associated with the surface area of the protein–ligand complex [85,86,87,88].

(4) $$\Delta E_{\mathrm{SURF}}=\gamma \sum^{i}\frac{1}{r_{i}},$$

where γ is a constant that depends on the solvent dielectric constant and ionic strength, and *r_i_* is the distance from atom *i* to the center of the solvent-accessible surface. The equation is based on the solvent-accessible surface area (SASA) model.

ΔG_GAS_: This term represents the Gibbs free energy change associated with the gas phase [89].

(5) $$\Delta G_{\mathrm{GAS}}=H-\mathrm{TS},$$

where H is the enthalpy, T is the temperature, and S is the entropy. The equation is derived from the Gibbs–Helmholtz equation.

ΔG_SOLV_: This term represents the Gibbs free energy change associated with the solvation of the protein–ligand complex [90].

(6) $$\Delta G_{\mathrm{SOLV}}=\Delta H_{\mathrm{SOLV}}-T\Delta S_{\mathrm{SOLV}},$$

where ΔH_SOLV_ is the enthalpy change associated with the solvation process, ΔS_SOLV_ is the entropy change associated with the solvation process, and T is the temperature. The equation is based on the thermodynamic definition of Gibbs free energy.

Δ_TOTAL_: This term represents the total energy change associated with the interaction of the protein–ligand complex with its environment [89,90].

(7) $$\Delta_{\mathrm{TOTAL}}\;=\;\Delta_{\mathrm{VDWAALS}}\;+\;\Delta E_{\mathrm{EL}}\;+\;\Delta E_{\mathrm{GB}}\;+\;\Delta_{\mathrm{ESURF}}\;+\;\Delta G_{\mathrm{SOLV}},$$

where each term is calculated using the appropriate equation as described above. The total energy change represents the overall stability or instability of the protein–ligand complex in its environment.

## Figures and Tables

**Table 1:** Partial least squares (PLS) parameters for generating field-based models.

Factors	SD	R^2^	R^2^_CV_	R^2^ Scramble	F	*p*-Value	RMSE	Q^2^	Pearson-r
1	0.52	0.62	0.49	0.30	42.50	0.00	0.39	0.69	0.91
2	0.38	0.81	0.40	0.49	53.00	0.00	0.35	0.75	0.87
3	0.30	0.88	0.48	0.63	59.30	0.00	0.30	0.81	0.91
4	0.24	0.93	0.51	0.73	78.50	0.00	0.25	0.87	0.94

**Table 2:** Data for field-based fractions.

Factors	Steric	Electrostatic	Hydrophobic	H-Bond Acceptor	H-Bond Donor
1	0.579	0.071	0.161	0.131	0.059
2	0.514	0.078	0.22	0.155	0.034
3	0.491	0.087	0.213	0.182	0.028
4	0.47	0.092	0.206	0.203	0.029

**Table 3:** PLS parameters for generating atom-based models.

Factors	SD	R^2^	R^2^_CV_	R^2^ Scramble	Stability	F	*p*-Value	RMSE	Q^2^	Pearson-r
1	0.50	0.65	0.51	0.38	0.95	48.70	0.00	0.34	0.77	0.94
2	0.33	0.86	0.51	0.53	0.79	74.30	0.00	0.40	0.67	0.86
3	0.28	0.90	0.51	0.68	0.74	71.10	0.00	0.31	0.80	0.91
4	0.23	0.94	0.47	0.78	0.61	85.30	0.00	0.26	0.86	0.93

**Table 4:** Data for atom-based fractions.

Factors	H-Bond Donor	Hydrophobic/Non-Polar	Electron Withdrawal
1	0.042	0.753	0.205
2	0.018	0.813	0.169
3	0.02	0.818	0.162
4	0.029	0.804	0.167

**Table 5:** PHASE-generated numerous pharmacophore theories.

Model	Survival	Site	Vector	Volume	Selectivity	Num-Matched	Inactive	Adjusted	Sites	PhaseHypo
DHRRR1	5.88	0.83	0.99	0.76	2.02	19	2.27	3.61	8.85	8.55
DHRRR2	5.86	0.83	0.99	0.75	2.02	19	2.27	3.60	8.85	8.55
DHRRR3	5.86	0.82	0.99	0.76	2.02	19	2.28	3.58	8.85	8.55
DHRRR4	5.85	0.83	0.98	0.75	2.01	19	2.28	3.57	8.85	8.55
DHRRR5	5.83	0.80	0.98	0.74	2.04	19	2.27	3.56	8.85	8.55
DHRRR6	5.83	0.80	0.98	0.74	2.03	19	2.24	3.59	8.85	8.55
DHRRR7	5.81	0.82	0.99	0.72	2.01	19	2.21	3.60	8.85	8.55
DHRRR8	5.80	0.80	0.97	0.72	2.02	19	2.14	3.66	8.85	8.55
DHRRR9	5.76	0.82	0.97	0.69	2.01	19	2.09	3.67	8.85	8.55
DHRR10	5.37	0.83	0.99	0.76	1.51	19	2.22	3.14	8.85	8.55
DRRR11	5.34	0.93	0.99	0.80	1.34	19	2.23	3.11	8.85	8.55
DHRR1	5.33	0.81	0.99	0.75	1.50	19	2.26	3.07	8.85	8.55
DHRR2	5.34	0.83	0.98	0.75	1.50	19	2.23	3.11	8.85	8.55
DHRR3	5.35	0.83	0.98	0.75	1.51	19	2.25	3.10	8.85	8.55
DHRR4	5.34	0.88	1.00	0.72	1.47	19	2.16	3.18	8.85	8.55
DHRR5	5.35	0.87	1.00	0.74	1.46	19	2.22	3.13	8.85	8.55
HRRR1	5.33	0.83	0.99	0.76	1.48	19	2.70	2.63	8.85	8.55
HRRR2	5.32	0.83	0.98	0.76	1.48	19	2.76	2.57	8.85	8.55
DHRR5	5.34	0.81	0.99	0.76	1.49	19	2.24	3.10	8.85	8.55

**Table 6:** Metrics for the DDRRR 1 hypothesis evaluation.

Hypothesis	DHRRR_1
PhaseHypo Score	1.35
EF1%	2.3
BEDROC160.9	0.96
ROC	0.87
AUAC	0.74
Ave Outranking Decoys	4.29
Total Actives	17
Ranked Actives	17
Matches	4 of 5
Excluded Volumes	Yes

**Table 7:** Predicted activity via field-based and atom-based methods.

Reference pIC_50_ = 9.15	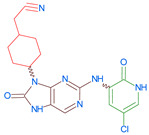		
ID	Two-Bimensional Compound Structure	Field-Based pIC_50_ (*Pred*)	Atom-Based pIC_50_ (*Pred*)
D1	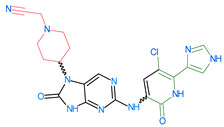	7.97	8.00
D2	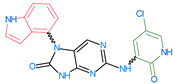	8.00	7.93
D3	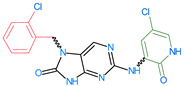	7.92	7.91
D4	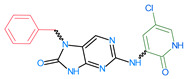	8.011	7.91
D5	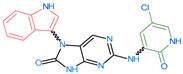	8.40	8.04
D6	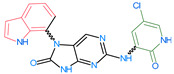	8.01	8.07
D7	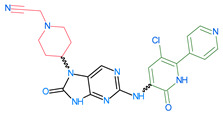	8.41	8.07
D8	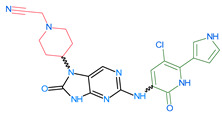	8.05	8.01
D9	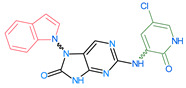	7.94	7.94
D10	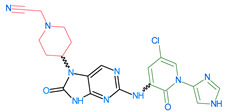	7.84	7.97
D11	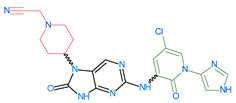	8.27	8.06
D12	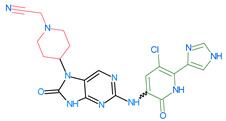	8.03	8.22
D13	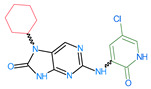	8.39	8.23

**Table 8:** ADMET analysis of the compounds that were newly designed.

ADMET	Rule	D1	D2	D3	D4	D5	D6	D7	D8	D9	D10	D11	D12	D13	Tofacitinib
LogS	−4–0.5	−3.582	−3.387	−3.928	−3.464	−3.861	−3.469	−3.941	−3.944	−3.463	−3.463	−3.388	−3.388	−3.872	−2.176
LogD	1–3	3.341	2.983	2.875	1.636	2.88	1.635	2.88	3.122	1.315	1.315	1.329	1.329	3.159	1.426
LogP	0–3	2.929	2.256	2.297	1.197	2.251	1.253	2.195	2.887	0.939	0.939	0.861	0.861	2.711	1.174
HIA	>30	0.849	0.64	0.938	0.42	0.853	0.797	0.848	0.155	0.252	0.252	0.462	0.462	0.438	0.934
Caco-2	>−5.15	−5.084	−5.132	−5.284	−5.829	−5.186	−5.824	−5.294	−5.17	−5.751	−5.751	−5.744	−5.744	−4.964	−4.655
MDCK	>20 × 10^−6^	1.33 × 10^−5^	1.07 × 10^−5^	4.10 × 10^−6^	7.24 × 10^−6^	3.84 × 10^−6^	4.97 × 10^−6^	4.26 × 10^−6^	8.33 × 10^−6^	4.48 × 10^−6^	4.48 × 10^−6^	5.18 × 10^−6^	5.18 × 10^−6^	5.59 × 10^−6^	6.3 × 10^−6^
BBB	0–0.3	0.041	0.027	0.012	0.107	0.011	0.073	0.01	0.009	0.35	0.35	0.073	0.073	0.037	
VDss	0.04–20	0.561	0.589	0.484	1.51	0.433	1.363	0.481	0.38	1.012	1.012	1.226	1.226	0.655	
1A2-inh		Yes	No	No	No	No	No	No	Yes	No	No	No	No	No	Yes
1A2-sub		Yes	Yes	Yes	Yes	Yes	Yes	Yes	Yes	Yes	Yes	Yes	Yes	Yes	No
2C19-inh		Yes	Yes	Yes	No	No	No	No	Yes	No	No	No	No	Yes	No
2C19-sub		No	No	No	No	No	No	No	No	No	No	No	No	No	No
2C9-inh		Yes	Yes	Yes	Yes	Yes	No	No	Yes	No	No	No	No	No	No
2C9-sub		No	No	No	No	No	No	No	No	No	No	No	No	No	No
2D6-inh		No	No	No	No	No	No	No	No	No	No	No	No	No	No
2D6-sub		No	No	No	No	No	No	No	No	No	No	No	No	No	No
3A4-inh		Yes	Yes	Yes	Yes	Yes	Yes	Yes	Yes	Yes	Yes	Yes	Yes	Yes	No
3A4-sub		No	No	Yes	Yes	Yes	Yes	Yes	No	Yes	Yes	Yes	Yes	No	No
CL	≥5	7.891	7.891	8.039	5.676	5.94	7.485	7.953	7.953	5.648	6.664	7.141	6.372	6.753	8.737
Ames		No	No	No	No	No	No	No	Yes	No	No	No	No	No	No

**Table 9:** The physicochemical features of the compounds that were newly designed.

P. P	nHA	nHD	TPSA	nRot	nRing	MaxRing	nHet	fChar	nStereo	MW	Lipinski
Rule	0~12	0~7	0~14	0~11	0~6	0~6	1~15	−4~4	≤2	100~600	Accepted
D1	8	3	111.69	3	4	9	10	0	0	402.040	
D2	8	3	111.69	3	4	9	9	0	0	368.080	
D3	9	4	127.48	2	5	9	10	0	0	393.070	
D4	11	3	151.61	4	5	9	12	0	0	477.140	
D5	9	4	127.48	2	5	9	10	0	0	393.070	
D6	11	4	154.51	4	5	9	12	0	0	465.140	
D7	9	4	127.48	2	5	9	10	0	0	393.070	
D8	9	3	116.62	2	5	9	10	0	0	393.070	
D9	12	3	156.54	4	5	9	13	0	0	466.140	
D10	12	3	156.54	4	5	9	13	0	0	466.140	
D11	12	4	167.4	4	5	9	13	0	0	466.140	
D12	12	4	167.4	4	5	9	13	0	0	466.140	
D13	8	3	111.69	2	4	9	9	0	0	360.110	
Tofacitinib	7	1	88.910	4	18	9	7	0	2	312.170	

**Table 10:** The affinity of compounds studied by CovDock.

Compound	D1	D2	D3	D4	D5	D6	D7	D8	D9	D10	D11	D12	D13	Tofacitinib
Affinity (Kcal/mol)	−9.10	−9.55	−9.37	−9.53	−9.5	−5.63	−6.71	−7–24	−6.36	−4.15	−7.42	−6.66	−8.10	−7.50

**Table 11:** Comparative study of delta energy (Kcal/mol) for the newly designed compounds D1–D5 in comparison with tofacitinib using MM/GBSA.

Delta Energy (Kcal/mol)	D1	D2	D3	D4	D5	Tofacitinib
Δ_VDWAALS_	−33.95	−36.93	−40.29	−32.60	−37.09	−22.82
ΔE_EL_	−30.27	−29.89	−33.86	−32.77	−19.55	−32.93
ΔE_GB_	42.36	46.61	48.34	45.61	34.48	55.89
ΔE_SURF_	−5.00	−4.78	−5.55	−4.78	−4.64	−3.34
ΔG_GAS_	−64.22	−66.82	−74.15	−65.37	−56.64	−55.75
ΔG_SOLV_	37.35	41.83	42.79	40.84	29.84	52.55
Δ_TOTAL_	−26.87	−24.99	−31.37	−24.54	−26.80	−3.20

**Table 12:** A comparative analysis of the experimental and predicted pIC50 values utilizing 3D-QSAR, field-based, and atom-based models.

Model 3D-QSAR				Field-Based	Atom-Based
No.	Compound	pIC_50_ (*Exp*)	QSAR	pIC_50_ (*Pred*)	
1	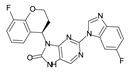	8.77	training	9.18	9.25
2	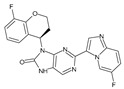	8.59	training	8.58	8.53
3	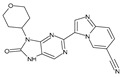	7.47	training	9.12	9.00
4	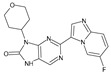	7.41	test	8.68	8.46
5	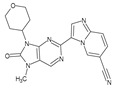	7.28	test	8.84	9.02
6	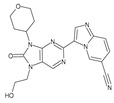	7.23	training	8.53	8.52
7	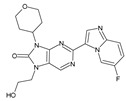	7.31	training	8.66	8.45
8	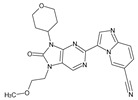	7.33	training	8.51	8.88
9	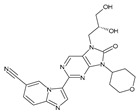	7.10	test	8.55	8.60
10	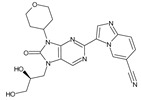	7.02	training	8.42	8.59
11	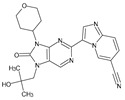	7.10	training	8.68	8.39
12	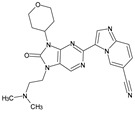	6.31	training	8.18	8.24
13	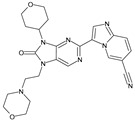	6.59	training	8.49	8.25
14	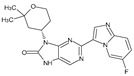	8.31	training	8.73	8.63
15	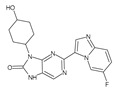	8.08	test	8.39	8.43
16	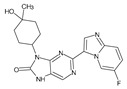	7.19	training	8.46	8.60
17	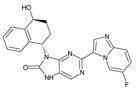	9.00	training	8.13	8.33
18	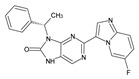	6.38	training	8.31	8.05
19	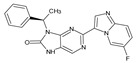	8.57	test	8.14	8.14
20	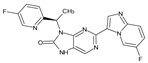	8.52	training	7.81	8.24
21	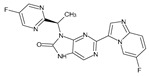	8.41	training	7.39	7.55
22	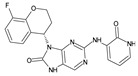	8.55	training	7.42	7.39
23	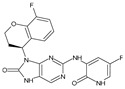	8.96	training	7.72	7.51
24	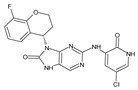	8.96	training	8.09	7.91
25	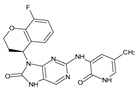	8.62	test	7.23	7.34
26	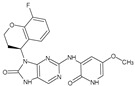	7.80	training	7.71	7.46
27	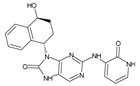	8.47	training	7.25	7.29
28	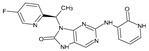	7.40	training	7.39	7.29
29	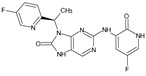	8.22	training	7.42	7.41
30	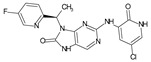	8.85	training	7.15	6.98
31	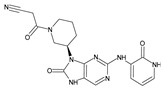	8.44	test	7.30	7.06
32	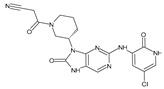	8.85	training	7.11	6.92
33	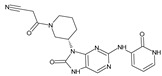	7.74	training	6.38	6.56
34	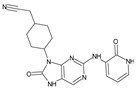	8.46	training	6.38	6.34
35	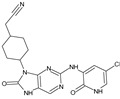	9.15	training	6.30	6.37

## Data Availability

Data are contained within the article.
